# Matrix metalloproteinases and their inhibitors in pediatric severe acute pancreatitis

**DOI:** 10.1371/journal.pone.0261708

**Published:** 2022-02-14

**Authors:** David S. Vitale, Patrick Lahni, Lindsey Hornung, Tyler Thompson, Peter R. Farrell, Tom K. Lin, Jaimie D. Nathan, Hector R. Wong, Maisam Abu-El-Haija

**Affiliations:** 1 Department of Pediatrics, University of Cincinnati College of Medicine, Cincinnati, Ohio; 2 Division of Gastroenterology, Hepatology and Nutrition, Cincinnati Children’s Hospital Medical Center, Cincinnati, Ohio; 3 Division of Critical Care Medicine, Cincinnati Children’s Hospital Medical Center, Cincinnati, Ohio; 4 Division of Biostatistics and Epidemiology, Cincinnati Children’s Hospital Medical Center, Cincinnati, Ohio; 5 Division of Pediatric General and Thoracic Surgery, Cincinnati Children’s Hospital Medical Center, Cincinnati, Ohio; 6 Department of Surgery, University of Cincinnati College of Medicine, Cincinnati, Ohio; University of Szeged, HUNGARY

## Abstract

**Background:**

Acute pancreatitis (AP) is increasing in incidence in adult and pediatric patients. Identification of patients at high risk for progression to severe acute pancreatitis (SAP) is crucial, as it can lead to increased mortality and health system cost. Matrix metalloproteinases (MMPs) are endopeptidases which degrade extracellular matrix proteins and increase activity of pro-inflammatory cytokines. Tissue inhibitors of metalloproteinases (TIMPs) regulate MMP activity. Prior limited studies of MMPs and TIMPs have found some to be associated with development of SAP. The aim of this study was to further investigate the role of MMPs and TIMPs in detecting pediatric patients at risk for developing moderately severe AP or SAP.

**Methods:**

Plasma samples were prospectively collected for patients <21 years of age presenting with AP between November 2015 and October 2019, along with healthy controls. Bead-based multiplex assays were utilized to test levels of 12 MMPs and TIMPs.

**Results:**

Samples were collected from 7 subjects who developed SAP, 7 with moderately severe AP, 45 with mild AP and 44 healthy controls. MMP-9 (p = 0.04) and TIMP-1 (p = 0.01) levels were significantly higher in SAP patients. A multivariable logistic regression model using MMP-9 and TIMP-1 predicted SAP (AUROC 0.87, 95% CI 0.76–0.98).

**Conclusion:**

We have demonstrated that MMP9 and TIMP1 levels are increased at AP presentation in pediatric patients who developed SAP during the course of illness. Further studies are needed to validate the use of MMPs and TIMPs as predictive tools for development of SAP in pediatric pancreatitis.

## Introduction

Historically, acute pancreatitis (AP) was believed to be uncommon in pediatrics, however the incidence of pancreatitis has been increasing [1, 2]. Nonetheless, pediatric AP has not been well investigated, and relevant data and guidelines are often extrapolated from adult literature. The natural course of patients who develop AP can range from a mild presentation without recurrence to severe acute pancreatitis (SAP), associated with significant morbidity and mortality [3]. Up to 15–34% of pediatric patients with AP have severe disease [2, 4]. Previously in pediatric patients, SAP has been defined by criteria including ICU admission, local complications, respiratory complications, need for surgical intervention and death [5]. In 2017, the North American Society for Pediatric Gastroenterology, Hepatology and Nutrition (NASPGHAN) Pancreas Committee published a new classification of pediatric mild AP, moderately severe AP and severe acute pancreatitis (SAP) [6].

Early identification of pediatric patients at higher risk for developing SAP is crucial. SAP is associated with increased health system costs and increased morbidity and mortality [3, 7]. Risk stratification of these patients early in their presentation allows for proper identification of patients requiring closer monitoring, higher level care or transfer to facilities more equipped to deal with consequences of SAP. In the future, these high-risk patients could be identified for targeted therapies or therapeutic trials, leading to implementation of a more personalized, patient-based care plan based on risk assessment.

Matrix metalloproteinases (MMPs) are endopeptidases which degrade extracellular matrix proteins. MMPs have been shown to be promoters of inflammatory processes by increasing activity of pro-inflammatory cytokines [8]. Twenty-three of these MMPs have been defined to date and are secreted by cells into the extracellular space, with activity regulated by their tissue inhibitors (TIMPs) [9].

Prior adult studies have analyzed the role of MMPs and TIMPs in development of SAP [10–15]. MMPs have been shown to be associated with acute and chronic pancreatitis, SAP and pancreatic necrosis [12–16]. Currently, there are no studies examining pancreatitis, MMPs and TIMPs in the pediatric population.

The aim of the present study is to evaluate MMPs and TIMPs as biomarkers for early identification of patients at risk for developing moderately severe AP or SAP. Data generated from this study can shed light on inflammatory pathways in SAP and help design possible therapies in the future. Based on previously reported adult data, we hypothesized that MMPs and their TIMPs would be useful prognostic biomarkers to aid in early risk stratification of patients presenting with AP.

## Methods

### Subjects

This study was conducted at a single institution, with a prospective cohort design. Patients were initially identified prospectively with their first episode of AP at Cincinnati Children’s Hospital Medical Center (CCHMC; Cincinnati, Ohio, USA) and enrolled in an AP registry (IRB 2012–4050) after written informed consent was obtained from the patient (>18 years of age) or parent/guardian (if patient <18 years of age). Patients <21 years of age were recruited for the study. Prior publications have included a subset of subjects in this study with different goals and objectives [7, 17–19]. Enrollment for this study occurred between November 2015 and October 2019. Criteria for diagnosis of AP were defined based on International Study Group of Pediatric Pancreatitis: In Search for a Cure (INSPPIRE) criteria and a more recent update from the European Pancreatic Club in collaboration with the Hungarian Pancreatic Study Group [20, 21]. Definition of AP was inclusive of two of the following three criteria: 1. Characteristic abdominal pain of pancreatic origin, 2. Lipase and/or amylase at least 3 times the upper limit of normal, and 3. Imaging (computed tomography, transabdominal ultrasound or magnetic resonance cholangiopancreatography) findings consistent with pancreatitis. Classification of AP attacks as severe, moderately severe or mild AP was defined by the North American Society for Pediatric Gastroenterology, Hepatology and Nutrition (NASPGHAN) clinical guidelines [6]. Age-matched control subjects were recruited from patients undergoing elective outpatient surgical procedures. These subjects had no history of prior pancreatic disease.

### Sample collection and assays

Blood samples were collected in EDTA tubes within 48 hours of admission for all participating subjects. Plasma was extracted from samples, aliquoted and stored at -80°C. MMP and TIMP plasma protein concentrations were subsequently measured using magnetic bead-based multiplex assays (Research and Diagnostic Systems, Inc., Minneapolis, MN) to test levels of MMPs 1, 2, 7, 8, 9, 10, 12, 13 and TIMPs 1, 2, 3, and 4. Immunoassays were conducted according to manufacturers’ specifications. Samples were diluted as appropriate per protocol. For each cytokine under investigation, eight control samples were run in duplicate to create a standard curve that was used to calculate the concentration of each cytokine. MMP-12, 13 and TIMP 3 levels were not detectable on the standard curve and therefore excluded from analysis. Values that fell outside the standard range but still on the standard curve were extrapolated.

### Statistical analysis

Data were analyzed using SAS®, version 9.4 (SAS Institute, Cary, NC). Due to small numbers and skewed distributions, continuous data were summarized as medians with interquartile ranges (IQR: 25^th^-75^th^ percentiles) while categorical data were summarized as frequency counts and percentages. For continuous data, non-parametric Kruskal-Wallis tests were used to compare characteristics and MMP/TIMP values between groups. Dwass, Steel, Critchlow-Fligner (DSCF) multiple comparison analyses were done to account for pairwise testing between two-sample Wilcoxon comparisons. Chi-square and Fisher’s exact tests were used, as appropriate, for group comparisons of categorical data. Logistic regression models were used to analyze the prediction of developing SAP and produce Receiver Operating Characteristics (ROC) curves. A *p*-value <0.05 was considered statistically significant.

## Results

Fifty-nine subjects met inclusion criteria of first attack AP and were recruited for participation in the study. Forty-eight of the 59 patients had samples collected <24 hours from presentation. Seven patients (12%) developed moderately severe AP, 7 (12%) developed SAP and 45 patients (76%) developed mild AP. There was no significant difference in age, sex, race, body mass index, weight or etiology of pancreatitis between the groups (Table 1). Comparison of controls to mild, moderate and severe AP showed significantly higher MMP and TIMP levels in AP patients for MMPs 1, 7, 8 and TIMPs 1 and 4 (Table 2).

**Table 1:** First acute pancreatitis attack.

	Severe AP	Moderate AP	Mild AP	P-value
	N = 7	N = 7	n = 45	
**Age 1**^**st**^ **AP attack (years)**	11.1 (7.7–14.7)	15.7 (8.3–18.7)	14.0 (10.6–15.3)	0.53
**Sex (male)**	6 (86%)	4 (57%)	20 (44%)	0.16
**Race**				0.26
** White/Caucasian**	6 (86%)	4 (57%)	37 (82%)	
** Black/African American**	1 (14%)	3 (43%)	5 (11%)	
** Other**	0 (0%)	0 (0%)	3 (7%)	
**BMI percentile**	74.9 (53.9–96.6)	36.9 (22.8–97.0)	59.9 (32.7–97.1) *n = 43*	0.77
**Weight percentile**	63.2 (7.0–94.6)	37.6 (23.9–96.5)	55.4 (14.6–87.0)	0.93
**Etiology**				0.21
** Divisum/Obstructive**	1 (14%)	0 (0%)	2 (4%)	
** Biliary/Gallstones**	2 (29%)	1 (14%)	6 (13%)	
** Genetic**	0 (0%)	1 (14%)	7 (16%)	
** Genetic/gallstones**	0 (0%)	0 (0%)	1 (2%)	
** Trauma**	0 (0%)	1 (14%)	0 (0%)	
** Systemic/Metabolic/Toxic**	2 (29%)	2 (29%)	9 (20%)	
** Drug induced**	0 (0%)	0 (0%)	6 (13%)	
** Drug + Other**	1 (14%)	2 (29%)	2 (4%)	
** Idiopathic**	1 (14%)	0 (0%)	12 (27%)	

Data presented as median (25^th^-75^th^ percentile) or n (%). AP = acute pancreatitis. BMI = body mass index.

**Table 2:** MMPs and TIMPs: SAP vs moderate AP vs mild AP vs controls.

	Severe AP N = 7	Moderate AP N = 7	Mild AP N = 45	Controls N = 44	P-value
**MMP 1**	11.2 (10.4–11.4)	11.4 (9.7–11.6)	10.8 (9.9–11.6)	9.9 (8.9–10.8)	**0.002**
**MMP 2**	16.1 (15.2–17.6)	16.5 (16.0–17.3)	16.4 (15.6–17.5)	17.9 (17.5–18.4)	**<0.0001**
**MMP 7**	9.7 (9.4–12.0)	10.2 (8.2–11.8) N = 1 estimated	10.0 (8.0–11.1), *n = 44* N = 7 estimated	8.0 (6.8–8.4), *n = 41* N = 12 estimated	**<0.0001**
**MMP 8**	13.4 (12.1–15.6)	11.6 (10.7–15.3) N = 1 estimated	13.1 (11.8–14.6) N = 6 estimated	10.7 (9.6–11.7) N = 17 estimated	**<0.0001**
**MMP 9**	18.6 (16.3–18.8)	14.6 (13.7–18.2)	16.0 (15.1–16.9)	16.2 (15.8–16.7)	0.07
**MMP 10**	9.1 (8.7–9.5)	8.6 (8.2–8.9)	9.2 (8.1–9.9) N = 2 estimated	8.8 (8.4–9.4) N = 1 estimated	0.34
**TIMP 1**	17.5 (17.1–18.4)	17.0 (16.7–17.8) N = 1 estimated	16.8 (16.4–17.3) N = 1 estimated	16.2 (16.0–16.4)	**<0.0001**
**TIMP 2**	16.7 (16.6–16.8)	16.6 (16.5–16.8)	16.5 (16.3–16.8)	16.7 (16.5–16.9)	0.14
**TIMP 4**	10.3 (9.5–11.1)	9.9 (9.8–11.4)	10.0 (9.2–10.4)	9.5 (9.1–9.9) N = 1 estimated	**0.008**

Data presented as median (25^th^-75^th^ percentile). AP = acute pancreatitis. MMP = matrix metalloproteinase. TIMP = tissue inhibitor of matrix metalloproteinase. SAP = severe acute pancreatitis.

*4 values for MMP 7 were missing due to being out of range.

**Fig 1:**
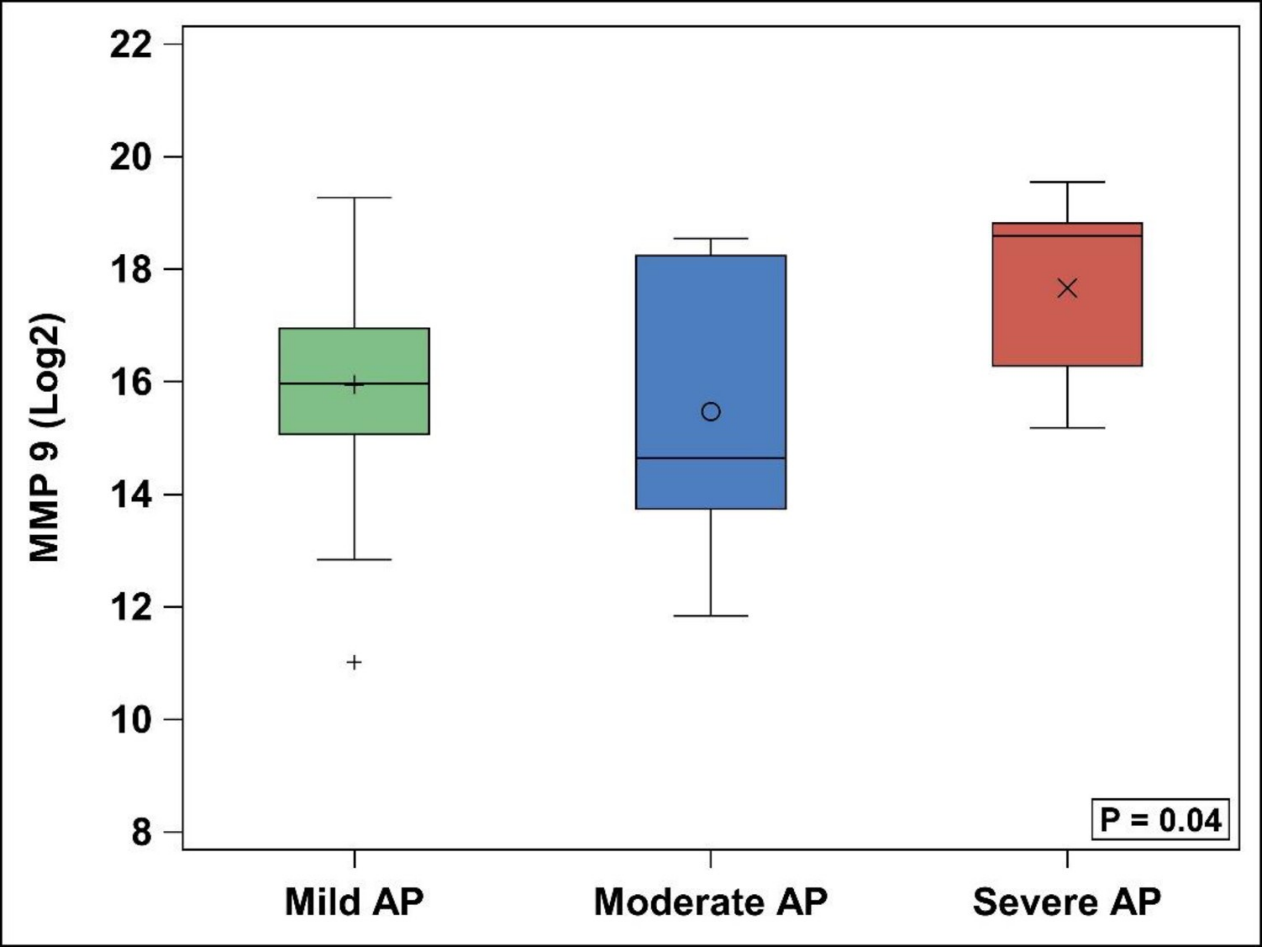
MMP-9 levels. AP = acute pancreatitis. MMP = matrix metalloproteinase.

**Fig 2:**
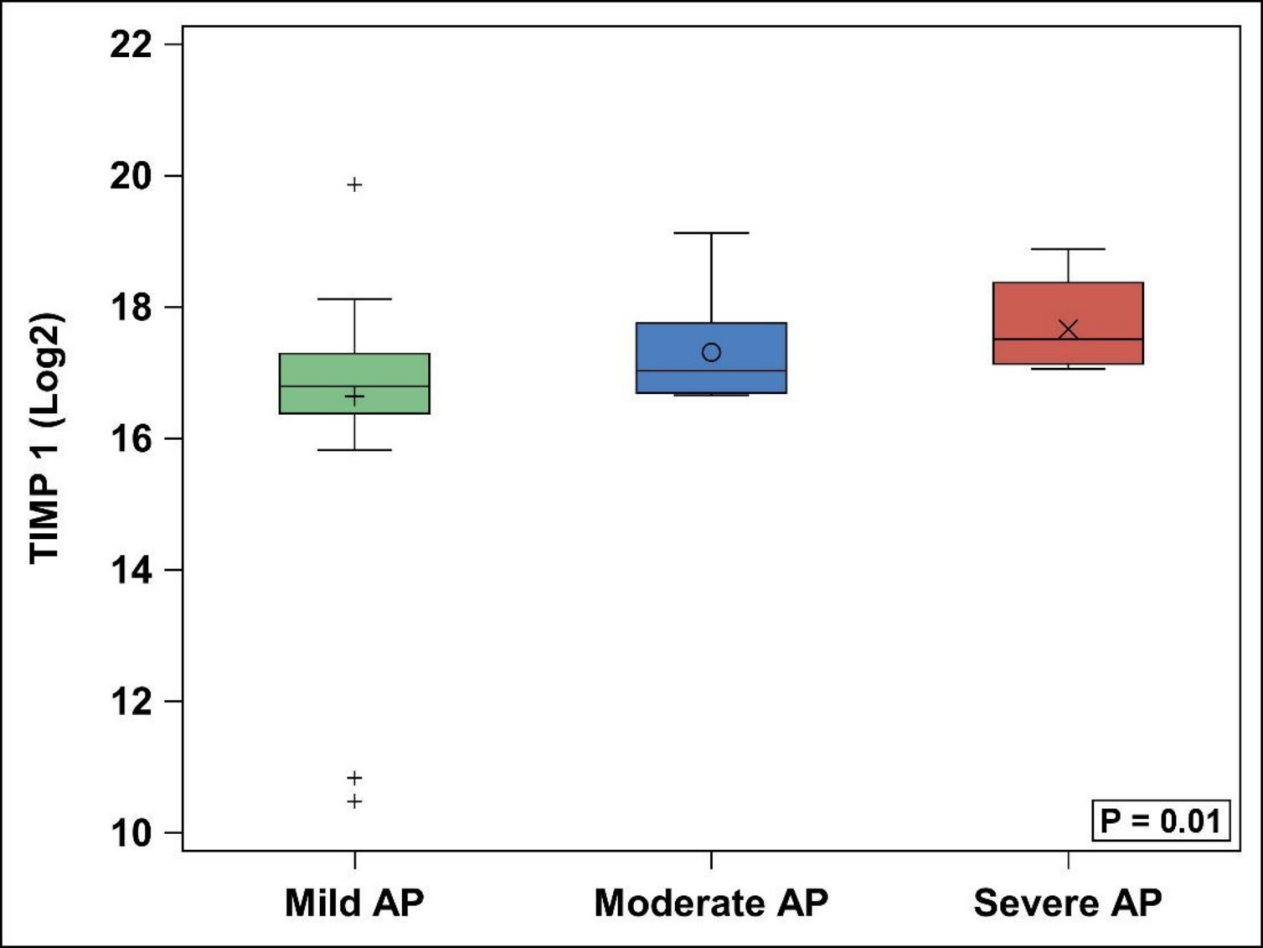
TIMP-1 levels. AP = acute pancreatitis. TIMP = tissue inhibitor of matrix metalloproteinase.

**Fig 3:**
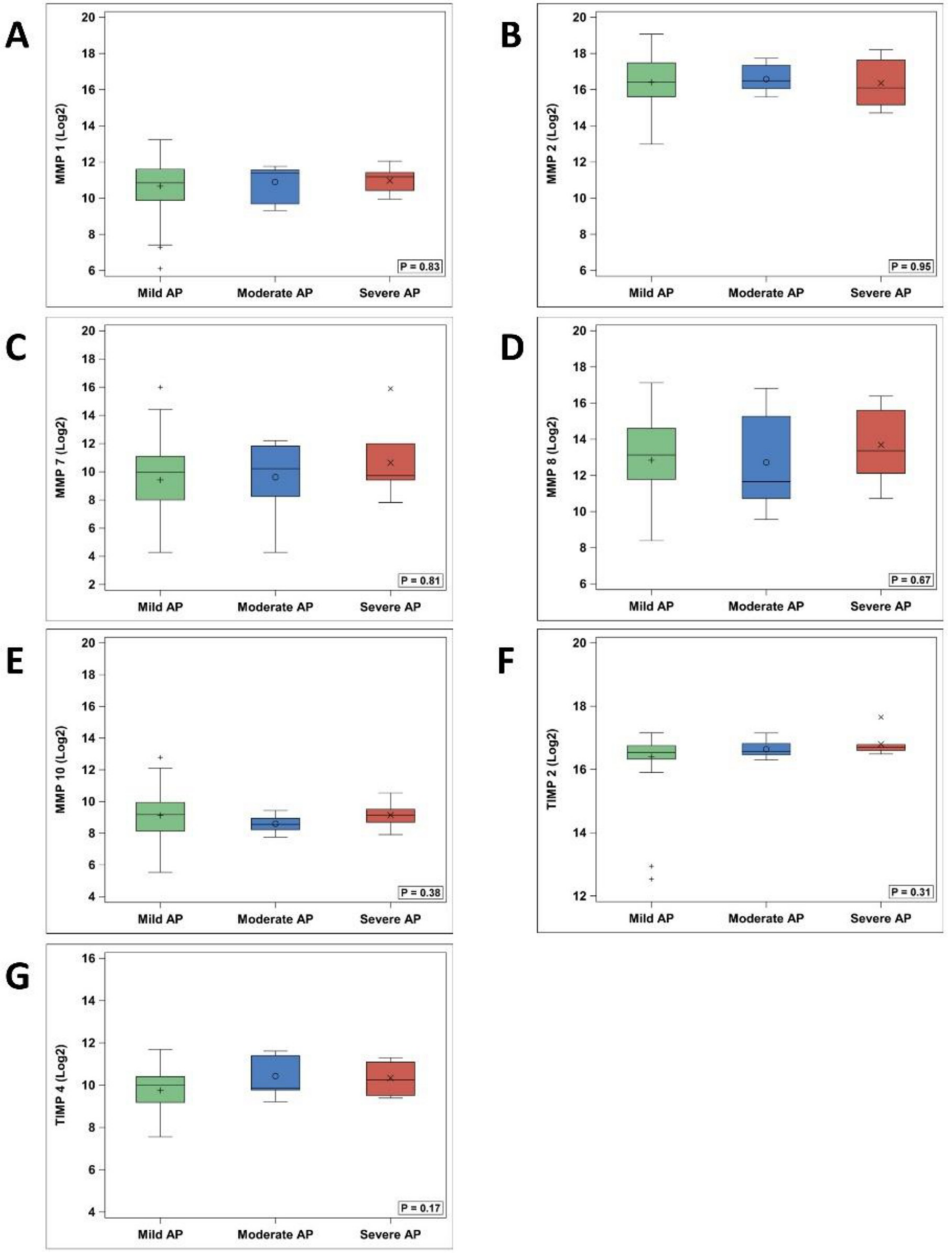
MMP-1, MMP-2, MMP-7, MMP-8, MMP-10, TIMP-2, TIMP-4 levels. AP = acute pancreatitis. MMP = matrix metalloproteinase. TIMP = tissue inhibitor of matrix metalloproteinase.

With comparison of mild AP, moderate AP and SAP groups, MMP-9 was found to be significantly different between the 3 groups (p = 0.04) (Table 3, Fig 1), with SAP patients having significantly higher median log MMP-9 levels (18.6, IQR: 16.3–18.8) than mild AP (16.0, 15.1–16.9) (p = 0.046). MMP-9 in moderate AP (14.6, 13.7–18.2) and SAP were not statistically different (p = 0.12), nor were mild and moderate (p = 0.79). Additionally, TIMP-1 was found to differ significantly amongst the three groups (p = 0.01) (Table 3, Fig 2), with direct comparison of mild AP (16.8, 16.4–17.3) and SAP (17.5, 17.1–18.4) showing higher TIMP-1 in SAP (p = 0.02). Similarly, TIMP-1 in moderate AP (17.0, 16.7–17.8) and SAP were not statistically different (p = 0.31), nor were moderate AP and mild AP (p = 0.38). The remainder of assayed MMPs and TIMPs, showed no significant differences between the three groups, or individually between SAP/mild AP and SAP/moderate AP (Fig 3).

**Table 3:** MMPs and TIMPs: Severe vs moderately severe vs mild AP.

	Severe AP N = 7	Moderate AP N = 7	Mild AP N = 45	P-value
**MMP 1**	11.2 (10.4–11.4)	11.4 (9.7–11.6)	10.8 (9.9–11.6)	0.83
**MMP 2**	16.1 (15.2–17.6)	16.5 (16.0–17.3)	16.4 (15.6–17.5)	0.95
**MMP 7**	9.7 (9.4–12.0)	10.2 (8.2–11.8) N = 1 estimated	10.0 (8.0–11.1) N = 7 estimated	0.81
**MMP 8**	13.4 (12.1–15.6)	11.6 (10.7–15.3) N = 1 estimated	13.1 (11.8–14.6) N = 6 estimated	0.67
**MMP 9**	18.6 (16.3–18.8)	14.6 (13.7–18.2)	16.0 (15.1–16.9)	**0.04**
**MMP 10**	9.1 (8.7–9.5)	8.6 (8.2–8.9)	9.2 (8.1–9.9) N = 2 estimated	0.38
**TIMP 1**	17.5 (17.1–18.4)	17.0 (16.7–17.8) N = 1 estimated	16.8 (16.4–17.3) N = 1 estimated	**0.01**
**TIMP 2**	16.7 (16.6–16.8)	16.6 (16.5–16.8)	16.5 (16.3–16.8)	0.31
**TIMP 4**	10.3 (9.5–11.1)	9.9 (9.8–11.4)	10.0 (9.2–10.4)	0.17

Data presented as median (25^th^-75^th^ percentile). AP = acute pancreatitis. MMP = matrix metalloproteinase. TIMP = tissue inhibitor of matrix metalloproteinase.

**Fig 4:**
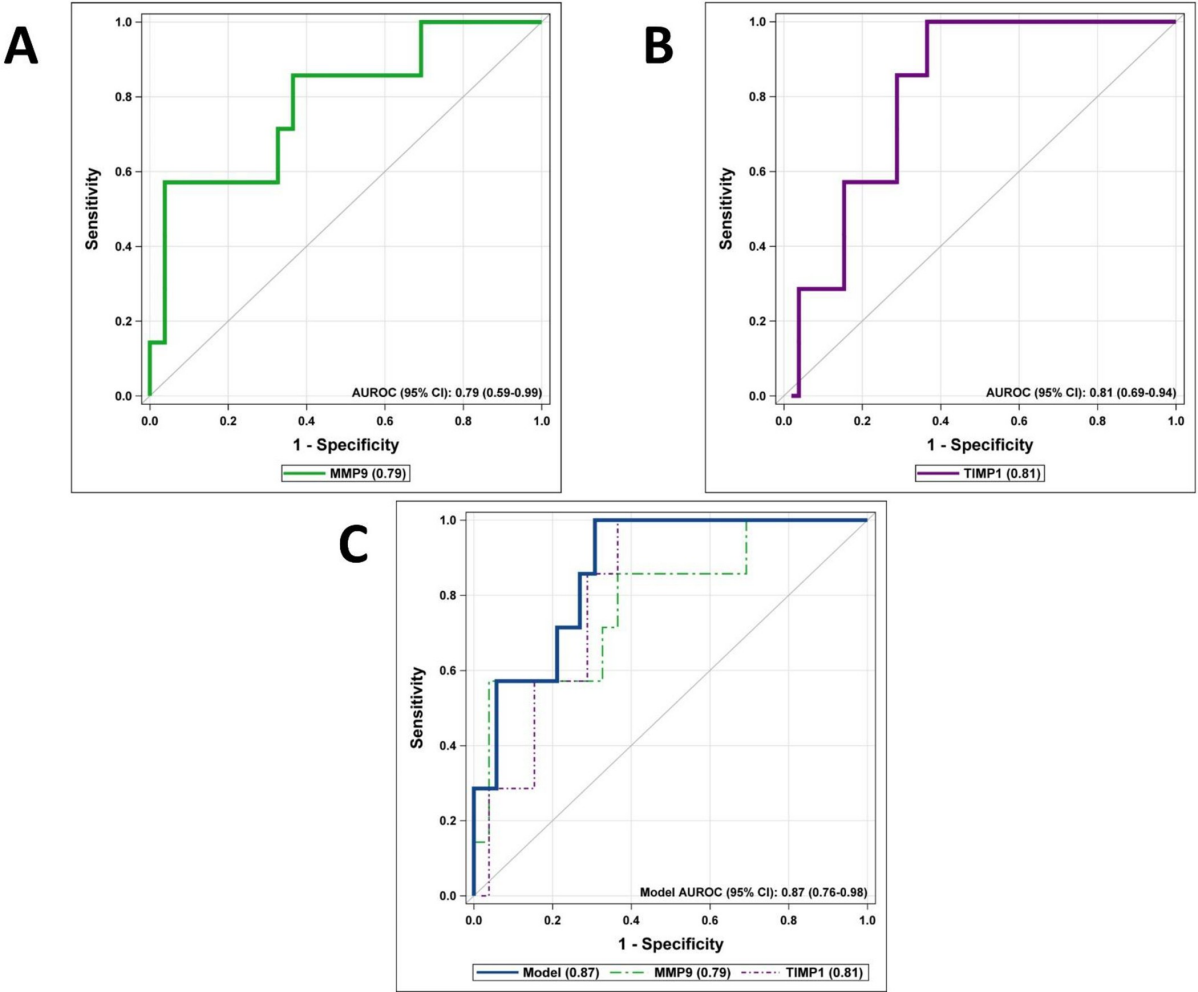
Detection of SAP using receiver operating curves (ROC). ROC curves demonstrating ability of MMP-9 (Fig 4A, p = 0.02) and TIMP-1 (Fig 4B, p = 0.04) to predict SAP. Fig 4C demonstrates a multivariable logistic regression model using both MMP-9 (p = 0.02) and TIMP-1 (p = 0.03) as predictors. SAP = severe acute pancreatitis. MMP = matrix metalloproteinase. TIMP = tissue inhibitor of matrix metalloproteinase.

Logistic regression models were generated and showed both MMP-9 (Fig 4A, AUROC 0.79, 95% CI 0.59–0.99, p = 0.02) and TIMP-1 (Fig 4B, AUROC 0.81, 95% CI 0.69–0.94, p = 0.04) to be significant predictors of SAP. A multivariable regression model utilizing both MMP-9 (p = 0.02) and TIMP-1 (p = 0.03) outperformed the individual models in predicting SAP (Fig 4C, AUROC 0.87, 95% CI 0.76–0.98).

## Discussion

This study evaluated MMP and TIMP levels in samples collected <48 hours after admission in a prospective pediatric cohort presenting with AP. It demonstrates increased levels of MMP-9 and TIMP-1 in patients who develop SAP, when compared to mild AP. This finding is novel in pediatric AP and presents future opportunities to investigate physiologic pathways involved in AP. There was no difference between moderate AP and SAP in any of the MMPs or TIMPs studied. Patients with AP were found to have significantly higher MMP-1, MMP-7, MMP-8, TIMP-1 and TIMP-4 levels than healthy control subjects. To our knowledge, this is the most extensive panel of MMPs and TIMPs studied in either adult or pediatric AP to date.

Attempts to identify predictors of SAP in pediatric AP have been ongoing [5, 7, 22–25]. Recent studies since the 2017 SAP criteria were published have utilized a uniform definition of pediatric SAP [7, 25], however prior studies with utilization of various criteria for SAP have not been able to be well disseminated or widely used. The importance of identifying biomarkers to predict risk for developing SAP cannot be overstated. High risk patients can be monitored more closely and/or transferred to higher level of care or facilities with expert care in managing SAP. Additionally, future studies may be useful in targeting therapies for patients at high risk for progression to more severe disease, as demonstrated in a recent study identifying patients with SAP and certain MMP gene mutations as better responders to a urinary trypsin inhibitor [26].

Similar to prior studies, our data showed higher levels of MMP-9 and TIMP-1 in patients developing SAP [14, 27]. In addition, in SAP, MMP-9 has been identified as an early marker of pancreatic necrosis [13], and possibly a susceptibility factor for chronic pancreatitis [15]. MMP-8, MMP-9 and TIMP-1 have been found to be increased in SAP in adults [14]. Recent adult studies have focused on MMP-2 and MMP-9, showing these as accurate single marker predictors of SAP with high specificity [27]. Gene polymorphisms in MMP-2 and MMP-9 were identified as indicators for efficacy of ulinastatin urinary trypsin inhibitor therapy for SAP [26]. In animal studies, MMPs have been shown to be released in relation to lung injury in AP [28]. and inhibition of MMP’s has been shown to decrease local and distant organ injury in pancreatitis [29].

MMP-9 is also known as gelatinase B and is secreted by neutrophils, macrophages and fibroblasts and degrades extracellular matrix. MMP-9 is inhibited by all TIMPs, which bind to the zymogen form of the enzyme, however TIMP-1 specifically inhibits active MMP-9 [30]. MMP-9 has previously been shown to be released in the inflammatory process in sepsis, cardiovascular disease and AP [11, 30–32]. Kinetics of MMP-9 have been studied with findings of serum peak at 6 or 12 hours, however these studies were in trauma and acute lung injury [33, 34]. While TIMP-1 is less well studied, it is known to be a direct inhibitor of MMP-9 [35] and has previously been speculated to be inadequately inhibiting MMP-9 in AP [10]. These prior studies and known functions of MMP-9 and TIMP-1 lend credence to the present study results with MMP-9 and TIMP-1 elevation in pediatric SAP. Kinetics of TIMP-1 in trauma patients have shown peak at 48 hours [34]. Further studies are needed, but our findings could lead to further insight into the pathophysiology of the inflammatory response in SAP and potentially lead to targeted therapies in the future.

Prior studies have also identified MMP-2 and MMP-8 as predictors of SAP, however, while our study showed a trend toward higher MMP-8 levels in SAP, it did not show a similar trend in MMP-2 levels. As expected, most of our studied MMPs and TIMPs were significantly higher in AP than controls, keeping with previously reported AP data. None of our patients with moderate AP demonstrated differences in MMPs or TIMPs between mild AP or SAP.

There are important strengths and limitations to our study. It was conducted at a single center and had a relatively small sample size. It is worth noting that limiting the study to first attack of AP only adds value to the design by eliminating many patient-related factors as variables. Our sample size compares favorably to existing pediatric AP literature. While other studies assessing MMPs and TIMPs have utilized even long periods of time from presentation for sample collection [14], our study used samples collected <48 hours from presentation. Based on the nature of our assays and median fluorescent index (MFI) results, some of the sample concentrations were extrapolated from the standard curve. While this is accepted practice in literature, this could lead to less accurate results and will need validation with protein level measurements in the future [36]. Lastly, as with all studies investigating AP, there is a limit to accurately identifying onset of pancreatitis as some patients present early in course of AP and others present after several days of symptoms. Despite these limitations, we feel this is a valuable initial investigation into MMPs and TIMPs as predictors of pediatric SAP.

In conclusion, our prospective cohort study in pediatric patients presenting with AP demonstrates increased MMP-9 and TIMP-1 levels in SAP when compared to mild AP. Data from our study can shed light on future targeted therapies in pediatric AP and progression to SAP. All patients with AP had higher levels of MMP-1, MMP-7, MMP-8, TIMP-1 and TIMP-4 than healthy controls. We developed a predictive prognostic model utilizing MMP-9 and TIMP-1. This represents the most comprehensive panel of MMPs and TIMPs studied in AP to date, and is an important first step in investigating MMPs and TIMPs as novel biomarkers of SAP in pediatric AP. Further studies evaluating the utility of MMPs and TIMPs are needed to validate and expand upon these results in patients with pediatric AP.
